# The Values, Acceptance, and Mindfulness Scale for Ice Hockey: A Psychometric Evaluation

**DOI:** 10.3389/fpsyg.2018.01794

**Published:** 2018-10-01

**Authors:** Tobias Lundgren, Gustaf Reinebo, Per-Olov Löf, Markus Näslund, Per Svartvadet, Thomas Parling

**Affiliations:** ^1^Centre for Psychiatry Research, Department of Clinical Neuroscience, Karolinska Institute, Stockholm, Sweden; ^2^Stockholm Health Care Services, Stockholm County Council, Stockholm, Sweden; ^3^Department of Psychology, Stockholm University, Stockholm, Sweden; ^4^MODO Hockey, Örnsköldsvik, Sweden

**Keywords:** ice hockey, psychometric, values, mindfulness, acceptance, VAMS

## Abstract

There is an increased interest in mindfulness, acceptance, and values based skills training interventions in sports but there is a lack of psychometrically evaluated instruments to investigate the processes adapted to sport populations. This paper describes the development and investigation of an instrument that measure acceptance, mindfulness, and values for ice hockey players. Ice hockey players at elite and sub elite level (*n* = 94) in Sweden participated in the study. The results reveal that the values, acceptance, and mindfulness (VAMS) shows acceptable internal consistency (α = 0.76) and satisfactory validity. Furthermore, scores on the VAMS predicts ice hockey performance as measured by assists and team points. Future research is suggested to evaluate the sensitivity of the instrument for longitudinal research design studies. In conclusion, VAMS is a useful instrument for practitioners and researchers to increase the knowledge in how psychological processes such as acceptance, mindfulness, and values influence performance among ice hockey players.

## Introduction

Mindfulness and acceptance-based methods were developed and adapted to sports inspired by the so called “third wave" of cognitive and behavior therapies (Gardner and Moore, 2004). This “third wave," also known as contextual cognitive and behavior therapy put focus on altering the relationship to private events rather than their frequency or form (Hayes et al., 2011). The theoretical rationale for these modern psychological interventions in sports therefore put more emphasis on mindful acceptance of private events while pursuing valued goals, in contrast to earlier forms of cognitive and behavioral therapies that included self-regulatory techniques (Gardner and Moore, 2004). However, the evidence for its effectiveness in sports is limited (Gardner and Moore, 2012; Buhlmayer et al., 2017) and instruments designed to investigate these processes adapted to sports are to our knowledge, non-existing. In a recent meta-analysis (Buhlmayer et al., 2017), investigating the effects found in randomized and non-randomized controlled studies with an active or inactive control group, the authors conclude that mindfulness and acceptance based programs may be beneficial for athletes and athletic performance. The authors found that indirect physiological and psychological performance variables such as flow, effective coping strategies, goal directed behavior, and maximized oxygen uptake are positively influenced following mindfulness training. Athletes are better equipped to cope with general anxiety and performance anxiety after mindfulness training. In addition, direct athletic performance was positively affected in precision sports such as dart throwing and shooting. Similar conclusions are drawn in another systematic review on mindfulness interventions in sports investigating single subject design studies as well as uncontrolled group trials (Sappington and Longshore, 2015). The authors suggest that there is preliminary support for mindfulness training for athletes both in regards to changes in indirect psychological and physiological performance variables as well as direct athletic performance. However, both the meta-analysis by Buhlmayer et al. (2017) and the systematic review by Sappington and Longshore (2015) suggest that the evidence is scarce. In general, there is a lack of well-designed, randomized controlled trials related to sport, especially with large samples using performance data as primary outcome measure. There is a wide range of sports where methodologically robust research on mindfulness procedures is not only scarce but also to our knowledge, non-existing. Furthermore, there is a lack of sport specific instruments to measure mindfulness, acceptance, and values in sports, which makes it difficult to investigate to what extent skills in these behavioral processes contribute to successful performance in elite athletes.

Context-specific measures are central to acquire knowledge on what processes that influence behavioral change in certain environments. Since mindfulness and acceptance based methods in sports are growing in popularity (Buhlmayer et al., 2017), there is a need to develop instruments that target mindfulness, acceptance, and values-based processes adapted to sport specific contexts. However, these processes have already been investigated in other domains of behavioral change. A commonly used instrument designed to target these processes in psychiatric settings as well as in organizational environments is the Acceptance and Action Questionnaire-II (AAQ-II) and the Work-related Acceptance and Action Questionnaire (WAAQ) (Bond et al., 2011, 2013; Lundgren and Parling, 2017). Scores on the AAQ-II predict life satisfaction outcomes such as depression, anxiety, and general mental health as well as future job satisfaction and job performance (Hayes et al., 2006). The AAQ-II has been used in sport studies to predict variables of interest, especially in sport rehabilitation. Mindfulness and acceptance skills as measured by the AAQ-II not only mediate adherence to rehabilitation procedures following sport injuries but also predict long term psychiatric problems for athletes (Baranoff et al., 2015; DeGaetano et al., 2016). AAQ-II appears to capture features important to sports and athletes and seems to be able to answer the questions of mindfulness and acceptance in a reliable manner (Zhang et al., 2014). However, the AAQ-II is not adapted to the sport context and the predictive characteristics regarding athletic performance are unknown. Population specific instruments seem to be better suited to detect important changes related to the population in focus (Gregg et al., 2007; Lundgren et al., 2008). To our knowledge there is no specific instrument developed to measure acceptance, mindfulness, and values processes in sports in general or specifically for ice hockey. Therefore, in order to enable adequate evaluations of psychological interventions that target mindfulness, acceptance, and values processes in ice hockey, the first step in our research endeavor is to develop such a measure.

The instrument is designed to capture three psychological processes hypothesized to be important in the context sports in general and ice hockey specifically; mindfulness, acceptance, and values. *Mindfulness* has been described in various ways in the literature but an often-used definition is the one by Kabat-Zinn (1994). Kabat-Zinn (1994) describes mindfulness as an act of being aware of the present moment in a non-judgmental, accepting, and non-avoidant way. In an ice hockey context mindfulness is defined as the ability to be consciously present and aware on the ice, direct attention according to what the situation demands and choose actions effectively during games and practice. *Acceptance* is an alternative to experiential avoidance and has been studied in length in clinical psychology and described in sports (Hayes et al., 1999; Gardner and Moore, 2004; Lundgren et al., 2008). Acceptance is described by Hayes et al. (1999) as an active and conscious embrace of private events occasioned by one’s history without the attempt to change their frequency or form. In an ice hockey context, acceptance is defined as an ability to actively embrace ice hockey related emotions, thoughts and memories in such a way that these experiences do not become obstacles to effective performance. *Values* significantly contribute to beneficial changes in outcomes such as life satisfaction and symptoms in various clinical treatment studies (Hayes et al., 2006; Lundgren et al., 2012). A value is often described as an individual verbal description of overarching life goals that add purpose, meaning, and motivation to actions in specific situations (Dahl et al., 2009). Values are not goals but rather guide and motivate goal driven behavior. Values in an ice hockey context is defined as the qualitative individual description of how ice hockey players want to approach games and practices in order to give themselves the best chance to be successful and enjoy their ice hockey.

The present study is part of a research collaboration between ice hockey organizations and the academia. The purpose of the collaboration is to develop psychological congruent measurement methods and training methods adapted for ice hockey players to enhance performance as well as investigating processes contributing to these potential effects. The aim of the present study is to develop and investigate a self-report instrument that measure values, acceptance, and mindfulness (VAMS) for ice hockey players. The research questions are (a) What is the component structure of the VAMS? (b) What is the internal consistency of the VAMS? (c) What is the content, concurrent, predictive and construct validity of the VAMS? (d) What is the relationship between the VAMS and performance as measured by assists, goals and team points?

## Materials and Methods

### Participants

Participants were recruited as part of the ice hockey project, a collaboration between MODO hockey, Stockholm University and the Karolinska Institute. All participants were ice hockey players, women (*n* = 20) and men (*n* = 73), playing at elite or sub-elite level in Sweden (Total *n* = 0.93). Participants consist of forward (*n* = 59), defenders (*n* = 27), and goalkeepers (*n* = 7). Demographic information is presented in **Table 1**. Participants were invited to participate in the study at a verbal presentation on sport psychology at the ice hockey organization. Study procedures was carried out in accordance with the Declaration of Helsinki, (World Medical Association General Assembly, 2004) reviewed, accepted and approved by the review committee at the Department of Psychology at Stockholm University, Sweden. All participants received written information about the study and returned a signed consent in accordance with the Declaration of Helsinki.

**Table 1:** Demographic features of the sample.

	*N (%)*	Age *M* (*SD*)
Women	20 (22)	20.5 (3.4)
Men	73 (78)	21.6 (4.7)
Total	93	21.4 (4.5)

### Material

All instruments used in the study are psychometrically evaluated in English and some in Swedish. To ensure the integrity of the Swedish translations the instruments were after translation back-translated to English and reviewed by an independent researcher. Instruments described below were administered to the participants in order to investigate the concurrent and construct validity of the VAMS. The measures were selected to cover a variety of perspectives related to processes of mindfulness, acceptance, values, and wellbeing.

The Swedish Acceptance and Action Questionnaire (SAAQ) (Lundgren and Parling, 2017) is a Swedish adapted version of the AAQ-II (Bond et al., 2011). SAAQ measures the concept of psychological flexibility (PF), which is the core psychological construct in acceptance and commitment therapy (ACT) and theoretically consists of mindfulness, acceptance and values processes (Hayes et al., 1999). The measure consists of 6 items. On a seven point Likert scale (1–7) participants rate how well they agree or disagree with statements such as “worries get in the way of my success.” Higher scores indicate lower PF and lower scores indicates higher PF. The AAQ-II correlates negatively with symptoms of depression, anxiety, and stress and positively with quality of life, general health, and job performance. The internal consistency of the SAAQ is satisfying (mean α = 0.85) and the test–retest reliability *r* = 0.80. In this sample α = 0.815.

The Depression, Anxiety, and Stress scale DASS-21 (Antony et al., 1998) is a short version of the DASS-42 (Lovibond and Lovibond, 1995). The DASS-21 measures symptoms of depression, anxiety, and stress over the past week on a 4-point Likert scale. Seven items per area measure depression, anxiety, and stress separately. Concurrent validity for the depression and anxiety subscales are moderately high. Internal consistency is satisfying for each subscale (depression α = 0.94, anxiety α = 0.87, and stress α = 0.91). Higher levels of stress as measured with the DASS 21 subscale are associated with lower levels of PF (Bond et al., 2011). Internal consistency for this sample; depression α = 0.82, anxiety α = 0.69, stress α = 0.82, and for the whole scale α = 0.90.

The Satisfaction With Life Scale (SWLS) (Diener et al., 1985; Pavot and Diener, 2008) measure Life satisfaction on a 7-point Likert scale. Participants rate how much they either agree or disagree on 5 items. An example of an item is *“in most ways my life is close to my ideal.”* The SWLS scale show satisfying internal consistency (α = 0.87) and good test–retest reliability (*r* = 0.82). Also, the SWLS scale correlate moderately to high with other instruments of quality of life. In this sample, internal consistency was α = 0.72.

Performance is measured in three ways (a) goals scored, (b) assists made, and (c) team points. Goals and assists refer to number of goals and assists made by an individual during the whole season. Team points refer to a summed score of total goals and assists while a player is on the ice but not necessarily scoring the goal or making the assists themselves, during the whole season. Data regarding performance was collected from elitprospect.com^1^.

### Procedure

The first author generated a pool of twenty-five items. All items were presented and discussed together with a group of four former National Hockey League (NHL) players. Items that the former players didn’t consider applicable were removed. When there were disagreements in the group regarding the validity of the items the first author had the final saying whether to keep or remove the item from the scale. This procedure resulted in 16 retained items. The items are rated on a 7-point Likert scale ranging between 1 = never true and 7 = always true. Scores are summed and can range between 16 and 112 points.

During a presentation on sport psychology at the organization, players were asked to read the information about the study and consider whether they wanted to participate. After consent was collected participants answered the questionnaires. The first author overlooked the administration process in order to be available to answer questions while a research assistant administered the instruments. The administration process took 30 min and was done in a conference room at the organization. Coaches or other members of the ice hockey organization were not present in the room while players were invited to participate in the study or answered the questionnaires. The questionnaires were administrated at the beginning of the season, September (2012) and the performance measures were collected at the end of the season, April (2013).

### Statistical Analysis

All measures were checked for outliers and normal distribution. In case of non-normal distribution non-parametric correlation was performed, otherwise Pearson correlation was used to investigate the validity of the VAMS. The attrition rate was low for all items and scales (between 0 and 0.07%). Mean value imputation were used to deal with missing data. Cronbach’s α was used to assess internal consistency. In case of non-normal distributed items on the VAMS instrument, the items were removed if they had an adverse effect on the following analysis, otherwise they were kept. Parallel analysis (PA) and Principal component analysis (PCA) was used to investigate the questionnaire. A PA was performed to determine the number of factors to extract (O’Connor, 2000). PA is indicated to be an accurate factor extraction (Horn, 1965; Zwick and Velicer, 1986). PCA were chosen as it is used for exploratory and reduction of data purposes. Emerging components were investigated with orthogonal or oblique rotation since components/subscales were considered theoretically correlated.

SPSS, SAS, MATLAB, and R Programs for Determining the Number of Components and Factors Using Parallel Analysis and Velicer’s MAP Test. We used the rawpar.sps syntax from the following website https://people.ok.ubc.ca/brioconn/nfactors/nfactors.html (downloaded on October 23, 2017).

## Results

In the result section we present the initial analysis of the 16-item scale, followed by the component structure of the 13-item version (**Table 2**) and finally the validity of the 11 item version.

**Table 2:** Component loadings (Pattern matrix) from the final PCA with oblimin rotation and eigenvalues, percent of variance, and internal consistency of the components for the VAMS.

Items	Components		
	Acceptance	Values	Mindfulness
1 I worry about not being able to control my nervousness	**0.746**	0.203	0.010
2 During games, I get nervous and I do not perform as well as I could	**0.744**	0.166	0.040
3 Worries get in the way of my success	**0.734**	−0.107	−0.078
4 It seems like most athletes are handling their feelings better than I am	**0.645**	−0.185	−0.099
5 If I fail in a shift I have a hard time letting it go and it affects me in upcoming shifts	**0.612**	−0.017	0.005
6 I long for games	0.041	**0.892**	0.011
7 I long for training sessions	0.089	**0.723**	0.110
8 I long to feel the thrill of the game	−0.148	**0.638**	−0.296
9 I always have a lot of time in the situations on the ice	−0.103	−0.061	−**0.888**
10 I always see different options in games and feel I have plenty of time to decide	0.126	0.168	−**0.841**
11 Even though I feel stressed during games I carefully weigh my decisions	0.181	−0.025	−**0.741**
Eigenvalue	3.35	1.84	1.51
% of variance	30.47	16.72	13.73
Cronbach’s α	0.75	0.63	0.82
*M* (*SD*)	13.56 (4.65)	6.89 (2.765)	10.34 (2.986)

**Table 3:** Concurrent and construct validity of the VAMS investigated with Pearson’s correlation coefficient.

	Values	Mindfulness	VAMS Total	SAAQ	DASS				SWLS
VAMS					tot	dep	anx	stress	
*n* = 94				*n* = 20	*n* = 39	*n* = 39	*n* = 39	*n* = 39	*n* = 38
Acceptance	0.10	0.34^∗∗^	0.81^∗∗^	0.69^∗∗^	0.49^∗∗^	0.45^∗∗^	0.31	0.46^∗∗^	−0.26
Values	–	0.24^∗^	0.54^∗∗^	0.11	0.19	0.27	0.09	0.13	−0.36^∗^
Mindfulness		–	0.71^∗∗^	0.24	0.16	0.11	0.25	0.04	−0.30
Total			–	0.61^∗∗^	0.47^∗∗^	0.45^∗∗^	0.36^∗^	0.38^∗^	−0.43^∗∗^

*^∗^p < 0.05, ^∗∗^p < 0.001. VAMS, values, acceptance and mindfulness scale for ice hockey; SAAQ, The Swedish Acceptance and Avoidance Questionnaire; DASS, Depression Anxiety and Stress Scale; SWLS, Satisfaction With Life Scale.*

The scores from the questionnaires (SAAQ, DASS-21, and SWLS) showed normal distributions without outliers. An initial check of the 16 items on the VAMS revealed one skewed item with an obvious floor effect and was therefore excluded. A first exploratory PCA indicated that the dataset was appropriate for further consideration (KMO = 0.647) and a significant Bartlett’s test of sphericity (*p* < 0.001). The PCA showed that 1 item showed a low anti-image coefficient (below 0.5) and was thus excluded. A PA on the remaining 14 items revealed a 3-component structure. The following PCA with three components as a criterion revealed that 2 items had communalities below 0.3. These 2 items were removed. Unrotated and rotated solutions showed that one additional item showed a high double loading (both with orthogonal and oblique rotation). Due to this the item was removed. The remaining 11 items were investigated in a PA and a 3-component structure was confirmed again. The following PCA resulted in higher variance explained and a satisfactory component structure with no alarming double loadings and all communalities above 0.3. For this final PCA the KMO was satisfactory (0.688), and Bartlett’s test of sphericity was significant (*p* < 0.001). Please see **Table 2** for the final component solution.

The VAMS total scale showed a Cronbach’s α = 0.76, with a mean = 33.46 (*SD* = 7.84) using a summed score (range 11–77). Participants were asked to rate how well they agreed or disagreed with the statements on the scale between 1 = never true and 7 = always true. Higher scores indicate higher ice hockey related PF. Scores on each individual item are summed. When calculating the summed score note that item 1–5 are reversed such that 1 equals 7, 2 equals 6 and so on. The remaining items are summed as they are.

The first component consists of 5 items that all assess the effect nervousness, possible failure, and worries have on the respondent’s performance. This component was named acceptance. The 3 items that loaded on the second component represent values since they are closely connected to purpose, meaning, and motivation to actions in specific situations. The last component with 3 items assess the degree of awareness of events while on the ice and was thus named mindfulness.

The predictive validity of the VAMS was investigated with Pearson’s correlation coefficient. The VAMS total scale was negatively associated with the performance measures Assist (*r* = −0.30, *n* = 59, *p* = 0.02) and Team Points (TP) (*r* = −0.29, *n* = 59, *p* = 0.028). The VAMS subscale mindfulness was significantly correlated with Assist (*r* = −0.38, *n* = 59, *p* = 0.003), and TP (*r* = −0.35, *n* = 59, *p* = 0.007). Number of goals was not significantly associated with the VAMS total or any subscale.

## Discussion

The outset of this study was to develop an instrument that measure the psychological processes acceptance, mindfulness, and values in ice hockey players, which is satisfyingly done with the VAMS. The VAMS is developed in collaboration between the academia and the sport field, which ensures face validity. Also, the VAMS show satisfactory internal consistency and good concurrent, construct and predictive validity. The VAMS significantly predicted direct ice hockey performance measures such as number of assists and TP. Furthermore, this study makes a psychometrically evaluated instrument available to sport researchers investigating acceptance, mindfulness, and values. Sport psychology practitioners interested in mindfulness and acceptance may use the VAMS to collect information on the effectiveness of their training procedures effect on these specific processes. Even though more research is needed to further investigate the relationship between the psychological processes measured in VAMS and performance the development of the VAMS has the potential to be a useful, psychometrically sound addition to sport psychology.

The VAMS, with 11 items, exhibited 3 subscales; values, acceptance, and mindfulness extracted from 16 items. The three subscales explained 61% of the variance and the items in each subscale did not show any double loadings. There were significant correlations between the three subscales and the total score. Also, our analysis revealed a significant correlation between the subscales Values – Mindfulness, Mindfulness – Acceptance but not between subscales Values – Acceptance. Internal consistency was adequate for the total VAMS and acceptable for the subscales for exploratory purpose (Garson, 2013).

The scale has good face validity since all items were discussed, explored, and chosen by professional ice hockey players and the first author. The majority of items are concrete and directly connected to the act of playing ice hockey. In addition, the mindfulness subscale and the VAMS total score showed significant predictive validity with the performance measures assist and TP. The total scale (and the acceptance subscale) showed good concurrent and construct validity due to its significant correlation with the SAAQ (acceptance and action). There were significant correlations between the VAMS and measures of emotional distress (depression, anxiety, and stress) which further strengthens the concurrent validity. These findings are also supported by earlier research investigating constructs with similar characteristics and their correlations with measures of emotional distress (e.g., Bond et al., 2011). The significant correlation between the values subscale of the VAMS and the SWLS is also supported by earlier research of these constructs in a Swedish context (SAAQ; Lundgren and Parling, 2017). Also, there is preliminary evidence of convergent validity since items load exclusively in the same component without cross-loadings (Garson, 2013).

Values, acceptance, and mindfulness give researchers the further opportunity to investigate mindfulness, acceptance, and values as potential mediators of efficient and non-efficient hockey behaviors. However, psychological constructs are reported and categorized observations of abstract psychological phenomena. The interpretation of what a mindful, accepting or values-oriented ice hockey player *really does* on the ice should therefore be well reflected in the items of the scale. This is also central to content validity. The first five items (see **Table 2**) refers to ice hockey player’s *acceptance* of private events, such as nervousness, worry, emotions, ruminations, and hesitations that can be obstacles to effective performance on the ice. High scores on these questions suggests that ice hockey players who can be open and embracing toward aversive private events, without trying to change their frequency or form, should be beneficial to ice hockey performance and in line with the theoretical description of acceptance (Hayes et al., 1999; Gardner and Moore, 2004). Items 6–8 reflects ice hockey players *values* with questions focusing on longing for training sessions, games and to feel the thrill of the game. Theoretically, values can be described as overarching goals, that guide goal driven behavior across situations (Dahl et al., 2009; Plumb et al., 2009). An interpretation of items 6–8 could be that ice hockey players who long to perform are also often in line with their values as athletes and how they want to approach performance and act on the ice. From a behavioral analytic point of view, behaviors that are guided by the individuals overarching goals and are intrinsically engaging is more likely to be positively reinforced and sustained as an effective part of the performing behavior repertoire, rather than behavior under aversive control (Plumb et al., 2009). Items 9–11 refer to how *mindful* behaviors could be manifested in the game. It describes players experiences of having enough time on the ice, making careful decisions even when they feel stressed, and if they are able to be aware of different options in the game, direct attention, and have the time they need to decide what play to go with. To optimally execute such performances on the ice, players needs to be fully engaged with the ongoing flow of unfolding events (both private and external) with a certain amount of dispassionate awareness, which is a typical description of mindful behavior (Kabat-Zinn, 1994; Fletcher and Hayes, 2005). Such a mindful approach on the ice gives enough (metaphorical) “space” to weigh different options and make adequate decisions in the ongoing game (context). As often described in ACT literature, some degree of present moment awareness is needed to behave consciously and deliberately, to live in concordance with your values, or to choose to be open and willingly embrace inner experiences as they are (Hayes et al., 1999). This is also central to a mindful, accepting, and values driven performance.

### A Couple of Limitations Warrants Attention

According to rules of thumb regarding number of participants per item for a PCA the sample size is on the border of small. However, in terms of statistical checks the dataset was appropriate for further analysis and the results of the PCA show a robust component structure with high loadings (0.61–0.88) without cross-loadings that suggest that the sample size is acceptable (Costello and Osborne, 2005). Also, the statistical relationship between the psychological processes measured in VAMS and performance needs further exploration. There is a significant correlation between VAMS and number of assists and also TP, not between VAMS and goals. A relationship between scored goals and the VAMS would be hypothesized but is not supported in this study. In order to clarify the relationship between performance and VAMS, further investigations are needed using larger samples.

### Strengths

The study has high ecological validity as the investigation took place in the natural habitat for the participants (inside the hockey organization) and that the instrument was developed together with ice hockey players. The sample consisted of elite and sub elite ice hockey players in Sweden, which lends support to external validity of the study.

An important next step for future research is to test the sensitivity of the scale when training ice hockey players. The aim of the present study was to develop an instrument that would be useful to the field of sport psychology. If training in VAMS can influence the ice hockey player’s scores on the VAMS it would strengthen the instrument as a valid outcome and process measure. Furthermore, investigations on how the scale predicts performance in players at the absolute highest level; the NHL and at levels below sub elite would also be of interest. Conducting such a study has the potential to investigate how components and items vary due to level of ice hockey expertise as well as the sensitivity to performance. Including the VAMS in longitudinal experimental designs where the measured processes are trained is an important future research endeavor to investigate the instruments sensitivity to change. Also, the instrument is developed in Swedish and the English version needs to be tested in an English-speaking environment.

## Conclusion

The VAMS is, to our knowledge the first instrument to measure acceptance, values and mindfulness processes in ice hockey players and have the potential to be a useful addition to sport psychology research. The VAMS shows initial satisfactory psychometrical properties and predicts not only psychological processes but also actual ice hockey performance. Due to the limited numbers of instruments developed for ice hockey players, the VAMS is a valuable addition to both practitioners and researchers in the field.

## Author Contributions

TL designed the study, developed the instrument, collected the data, contributed to data analysis, and wrote the manuscript. GR developed the instrument and wrote the manuscript. P-OL, MN, and PS developed the instrument, collected the data, and wrote the manuscript. TP designed the study, analyzed the data, and wrote the manuscript.

## Conflict of Interest Statement

The authors declare that the research was conducted in the absence of any commercial or financial relationships that could be construed as a potential conflict of interest.

## Footnotes

1 http://www.eliteprospects.com
